# Brassica napus Bacterial Assembly Processes Vary with Plant Compartment and Growth Stage but Not between Lines

**DOI:** 10.1128/aem.00273-22

**Published:** 2022-04-28

**Authors:** Jennifer K. Bell, Steven D. Mamet, Bobbi Helgason, Steven D. Siciliano

**Affiliations:** a Soil Science Department, College of Agriculture of Bioresources, University of Saskatchewan, Saskatoon, Saskatchewan, Canada; University of Illinois at Urbana-Champaign

**Keywords:** *Brassica napus*, assembly, canola, phyllosphere, rhizosphere

## Abstract

Holobiont bacterial community assembly processes are an essential element to understanding the plant microbiome. To elucidate these processes, leaf, root, and rhizosphere samples were collected from eight lines of Brassica napus in Saskatchewan over the course of 10 weeks. We then used ecological null modeling to disentangle the community assembly processes over the growing season in each plant part. The root was primarily dominated by stochastic community assembly processes, which is inconsistent with previous studies that suggest of a highly selective root environment. Leaf assembly processes were primarily stochastic as well. In contrast, the rhizosphere was a highly selective environment. The dominant rhizosphere selection process leads to more similar communities. Assembly processes in all plant compartments were dependent on plant growth stage with little line effect on community assembly. The foundations of assembly in the leaf were due to the harsh environment, leading to dominance of stochastic effects, whereas the stochastic effects in the root interior likely arise due to competitive exclusion or priority effects. Engineering canola microbiomes should occur during periods of strong selection assuming strong selection could promote beneficial bacteria. For example, engineering the microbiome to resist pathogens, which are typically aerially born, should focus on the flowering period, whereas microbiomes to enhance yield should likely be engineered postflowering as the rhizosphere is undergoing strong selection.

**IMPORTANCE** In order to harness the microbiome for more sustainable crop production, we must first have a better understanding of microbial community assembly processes that occurring during plant development. This study examines the bacterial community assembly processes of the leaf, root, and rhizosphere of eight different lines of Brassica napus over the growing season. The influence of growth stage and B. napus line were examined in conjunction with the assembly processes. Understanding what influences the assembly processes of crops might allow for more targeted breeding efforts by working with the plant to manipulate the microbiome when it is undergoing the strongest selection pressure.

## INTRODUCTION

Projected rapid increases in climate variability and global population (1) make the need for crops with resilient microbiomes ever more pressing (2). Canola (Brassica napus L.) is a globally important oilseed crop with high resource demands, making it an ideal target for microbiome engineering. Engineered microbiomes have the potential to increase disease resistance, enhance yield, and promote nutrient cycling (2). In addition to its high-quality oil, canola has been increasingly used as high-quality animal feed and to produce biofuels. However, canola requires large nitrogen inputs and is susceptible to common crop diseases like Fusarium wilt, both of which could be addressed through more targeted microbiome manipulations. Previous studies of canola-associated microbiomes focused primarily on the roots and rhizosphere (3–6) or specific microbial isolates (7), or it was not the primary focus of the overall study (8, 9). Microbiome-centered approaches increase plant tolerance to abiotic stresses, disease, and low nutrients (10, 11), though these benefits may be helped or hindered by microbial community assembly processes. Thus, a clear understanding of microbial community assembly is needed before we can create a sustainable microbiome that increases crop yield stability (12).

Two broad processes—deterministic and stochastic—influence community assembly of species (11). Deterministic processes are more directed and rely on ecological filters such as homogenizing (more closely related communities than expected by random chance) or heterogenous (more distantly related communities than expected) selection (12). Stochastic processes include dispersal events and drift or diversification (13) and are grouped into homogenizing dispersal and dispersal limitation. Dispersal refers to the movement of species from one habitat to another, and drift is the random division, death, ecological drift (random fluctuations in species abundance), or diversification (mutation) of individuals within a community (12, 13). Homogenizing dispersal includes high rates of dispersal between habitats leading to similar communities. Dispersal limitation can lead to high rates of community turnover and more dissimilar communities. Disentangling community assembly processes in microbial communities is essential to fully understanding how these communities function. For example, Ning et al. (14) found that homogeneous selection of soil microbiome in a grassland was correlated with drought and higher plant productivity under warmed conditions.

The relative influence of stochastic and deterministic processes in community dynamics vary through space and time (14–16). Productivity and resource availability (17) are among several factors that influence the relative importance of stochastic versus deterministic processes (18). As crop plants develop and alter their environment, it is reasonable to expect an increase in the relative influence of deterministic processes (19), as selective pressures filter the initial microbial community (14). If microbial communities can be linked to improved crop performance, crop development programs may be able to leverage the microbiome at specific stages of phenological development to improve plant performance. For example, Wagner et al. (20) found that in Boechera stricta (Drummond’s rockcress), microbes could alter plant flowering time—an important canola breeding target correlated with yield stability. Understanding how the community assembles before flowering would allow the potential manipulation of this community to optimize flowering time. A useful metric to disentangle community assembly processes is to use a null model framework based on the phylogeny of the microbial communities (12, 15–17). Microbial phylogenies are useful tools in understanding microbial communities because unlike most metrics, they preserve the genetic relationships between bacterial taxa, and many bacterial traits have been shown to be conserved (21). The phylogeny is repeatedly randomized to give a distribution of theoretical phylogenies that could occur if no selection processes were acting upon the community (17). If the observed phylogeny falls two standard distributions outside the mean null model distribution, then we can conclude that some selection process is acting upon the real community (17). This framework allows for a more accurate estimation of ecological processes shaping microbial communities.

We selected eight phenologically diverse founder lines of a B. napus nested association mapping (NAM) panel to evaluate if bacterial community assembly in plant organs could be altered via breeding programs. We hypothesized that (i) community assembly processes differ among plant structures due to habitat differences, (ii) assembly processes would vary with B. napus line, and (iii) the root surface and the leaves would have the strongest deterministic assembly processes leading to more homogeneous communities, whereas the rhizosphere would be dominated by stochastic community assembly processes leading to more heterogenous communities. The leaves, roots, and rhizosphere soil of eight lines of B. napus were sampled weekly over the course of 10 weeks beginning 3 weeks after planting when the plants were at the five- to six-leaf stage. All weeks after this are reported as weeks after planting (WAP). We then used a null model framework as well as ordination approaches to elucidate the assembly processes governing bacterial community assembly throughout the growing season.

## RESULTS

**FIG 1:**
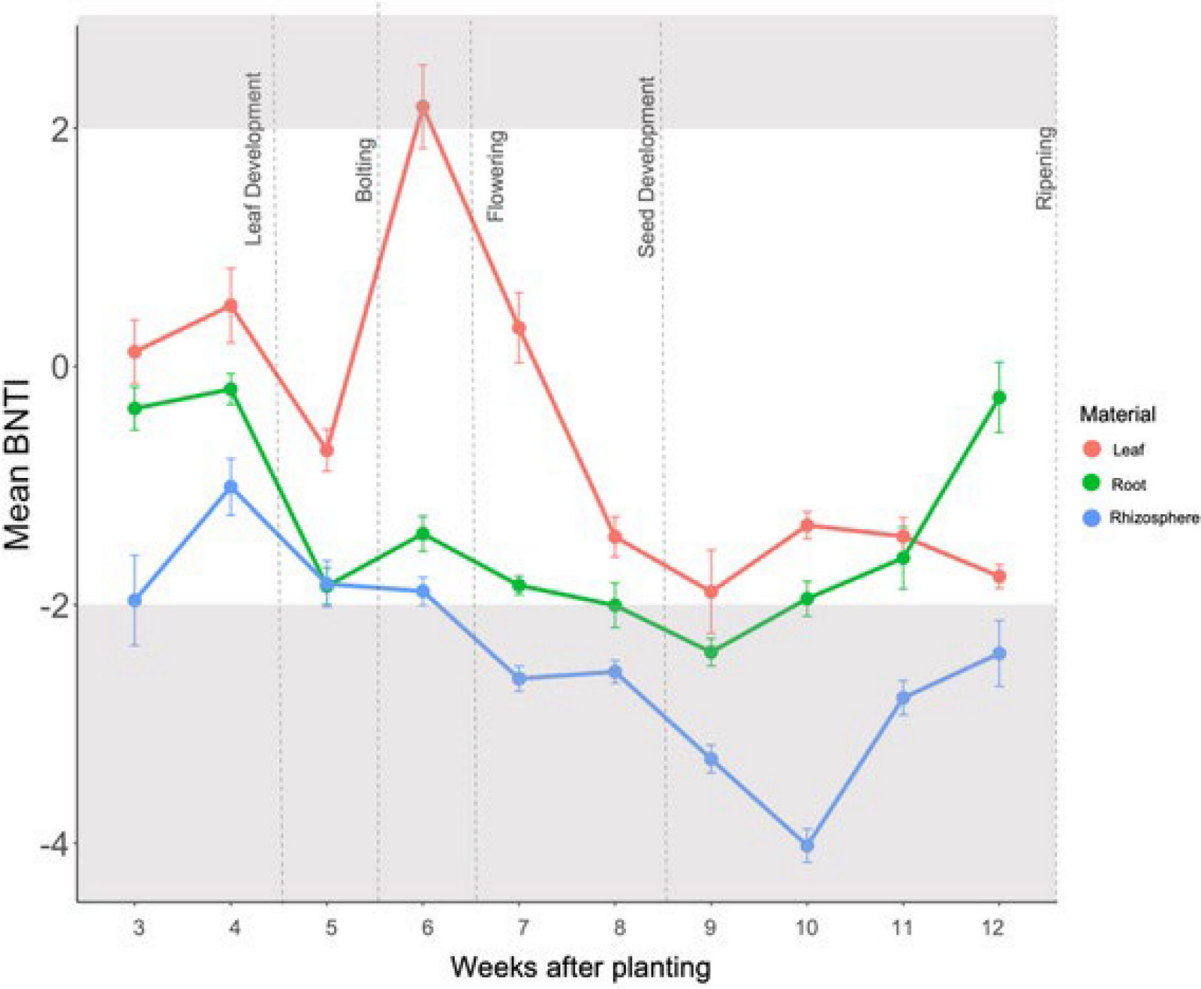
Mean βNTI for leaf, root, and rhizosphere samples over the 10-week sampling period. Each point represents 27 samples, and the error bars are the standard error. Growth stage is indicated by the dashed lines. Positive values indicate heterogenous selection is occurring, whereas negative values indicate homogeneous selection. The gray shaded area indicates a significant deviation from the null hypothesis.

Pielou’s evenness (Fig. 1) (22) was the lowest during flowering for both root and leaf. Interestingly, rhizosphere diversity was at its lowest during bolting but increased steadily after flowering. However, much like the leaf and root communities, Pielou’s evenness was the lowest for rhizosphere communities during flowering (Fig. 1; Table S1 in the supplemental material). Both the abundance-based coverage estimate (ACE) (23), and the Simpson index (24) for the leaf and root bacterial communities reached their peaks during weeks six and seven (25) or when the plants were flowering (Table S1).

Bacterial communities on the leaf, root, and rhizosphere were primarily composed of *Proteobacteria* with mostly *Gammaproteobacteria* (Fig. S1). In leaves, *Gammaproteobacteria* ranged from a high of 75% during week 9 to a low of 40% during week 12. In leaves, the second largest group consisted of classes not found in the root and rhizosphere communities (Fig. S1) but consisted primarily of *Bacteroidetes* (7%), *Acidobacteria* (6%), and *Firmicutes* (6%). In the root, *Gammaproteobacteria* comprised greater than 50% of the community in every week except week three. After *Gammaproteobacteria*, the dominant class in the roots was *Bacteroidia*. *Gammaproteobacteria* was also the dominant class in the rhizosphere, ranging from 30% during week three to 79% during week six (Fig. S1). Similar to the root communities, *Bacteroidia* was the second most dominant class present in the rhizosphere.

The influence of a Brassica napus line (NAM line) on bacterial community composition was inconsistent in each plant compartment and showed no clear trend throughout the growing season. Specifically, the NAM line was never a significant explanatory variable for leaf communities. For root bacterial communities, NAM line was a significant explanatory variable only during weeks four and seven (*P* = 0.01 and 0.001, *R*^2^ = 0.30 and 0.2, respectively). The NAM line was a significant explanatory variable for 6 out of the 10 sampling weeks for rhizosphere bacterial communities (*P* < 0.05, *R*^2^ = 0.23 to 0.32) (Table S3), but there was no consistent time period in which NAM line was or was not significant. Finally, even when the NAM line was significant, it rarely explained much of the variation (Table S2).

The growth stage (BBCH) was a consistent influence on all phylogenetic metrics (net relatedness index [NRI], nearest taxon index (NTI), and β-nearest taxon index [βNTI]). BBCH was a significant (*P* < 0.001) explanatory variable for NTI, NRI (Table 1), and βNTI (Table S2), demonstrating the influence of growth stage on bacterial assembly processes. Interestingly, the NAM line was significant for root NTI values (*P* = 0.03) but not for root NRI values or root βNTI values (Table S3). Similarly, the NAM line was significant for rhizosphere βNTI values but not rhizosphere NTI nor NRI values. There were no significant interactions between NAM line and BBCH growth stage for any compartment.

**TABLE 1:** Two-way ANOVA for the effect of B. napus line (NAM) and growth stage (BBCH) on the nearest taxon index (NTI) and net relatedness index (NRI) values for the leaf, root, and rhizosphere over the 10-week sampling period^*a*^

Metric or plant part	Degrees of freedom	Sum of squares	Mean of squares	*F* value	*P*
NRI					
Leaf					
NAM	7	0.66	0.0944	0.33	0.94
BBCH	32	22.53079.28	0.7268	2.539	<0.0001
Residuals	307		0.2862		
Root					
NAM	7	17	2.428	1.551	0.150343
BBCH	23	114	3.562	2.275	0.000219
Residuals	267	418	1.566		
Rhizosphere					
NAM	7	0.49	0.0706	0.205	0.984
BBCH	32	72.33	2.2604	6.545	<0.0001
Residuals	267	92.21	0.3453		
NTI					
Leaf					
NAM	7	8.1	1.154	0.725	0.651
BBCH	31	432.8	13.962	8.778	<0.0001
Residuals	277	440.6	1.59		
Root					
NAM	7	25.8	3.68	2.249	0.0308
BBCH	32	214.4	6.699	4.094	<0.0001
Residuals	267	436.9	1.636		
Rhizosphere					
NAM	7	6.11	0.873	1.596	0.137
BBCH	32	124.16	3.88	7.09	<0.0001
Residuals	267	146.12	0.547		

a There were no significant interactions, so they were not included in the final model.

**FIG 2:**
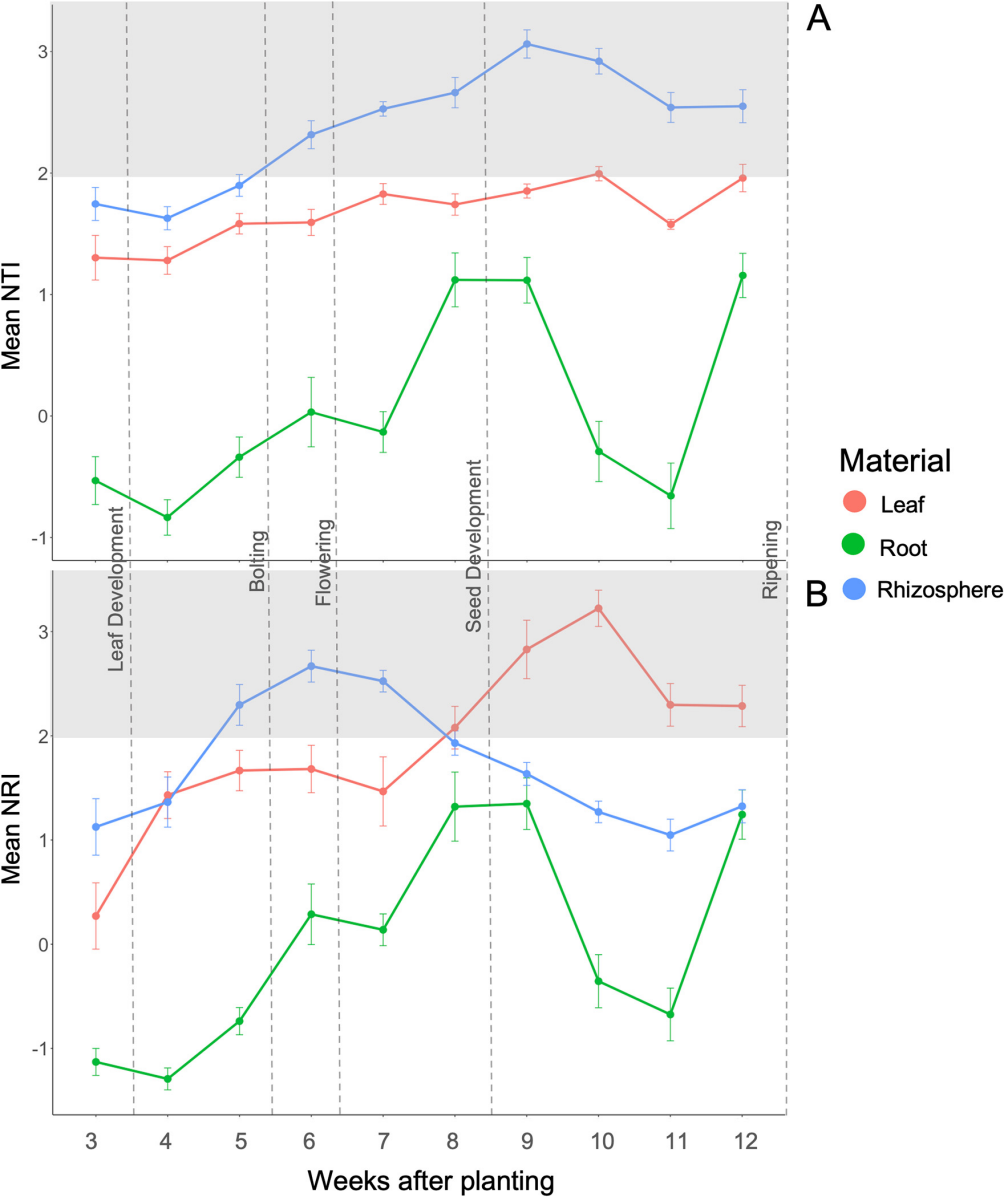
(A) Mean nearest taxon index (NTI) for leaf, root, and rhizosphere samples over the 10-week sampling period. (B) Mean net relatedness index (NRI) for leaf, root, and rhizosphere samples over the 10-week sampling period. Each point represents 27 samples, and the error bars are the standard error. Growth stage is indicated by the dashed lines. Positive values indicate more phylogenetic clustering than expected by chance, whereas negative values indicate phylogenetic overdispersion. The gray shaded area indicates a significant (*P* > 0.05) phylogenetic clustering compared to the null hypothesis.

The leaf communities were always more clustered phylogenetically than expected, especially after flowering, suggesting that there were selection pressures occurring during this period. Mean leaf NRI values were consistently greater than zero throughout the growing season, indicating an increasing trend of phylogenetic clustering leading to more similar communities as the growing season progressed (Fig. 2B) (26). However, leaf NRI values did not differ from the null hypothesis (|NRI| < 2; *P *> 0.05) until weeks 5 to 12 (*P *≤ 0.05), indicating that strong selection processes were not occurring. Leaf NTI did not differ from the null hypothesis until week nine (*P *≤ 0.05) (Fig. 2A).

In the root, no strong clustering or overdispersion was detected. Mean root NTI values were consistently different than zero, though they did not differ from the null hypothesis (*P* > 0.05) (Fig. 2A), meaning that strong selection was not occurring. Root NRI values showed similar trends as NTI values in that they were consistently greater than zero and did not differ from the null hypothesis (Fig. 2B). Despite the lack of strong selection pressures, BBCH (*P *< 0.001) was significant for both NTI and NRI values, and the NAM line (*P* = 0.0308) was significant for NRI values.

Rhizosphere NTI values showed stronger clustering of the bacterial communities than the rhizosphere NRI values. Rhizosphere NTI values were greater than zero and differed from the null hypothesis (*P *≥ 0.05) (Fig. 2A), which implies selection is occurring in this habitat. Rhizosphere NRI values were greater than zero but only differed from the null hypothesis in weeks six through nine (Fig. 2B). BBCH was significant (*P* < 0.001) for both rhizosphere NTI and NRI values, and the NAM line was not significant.

**FIG 3:**
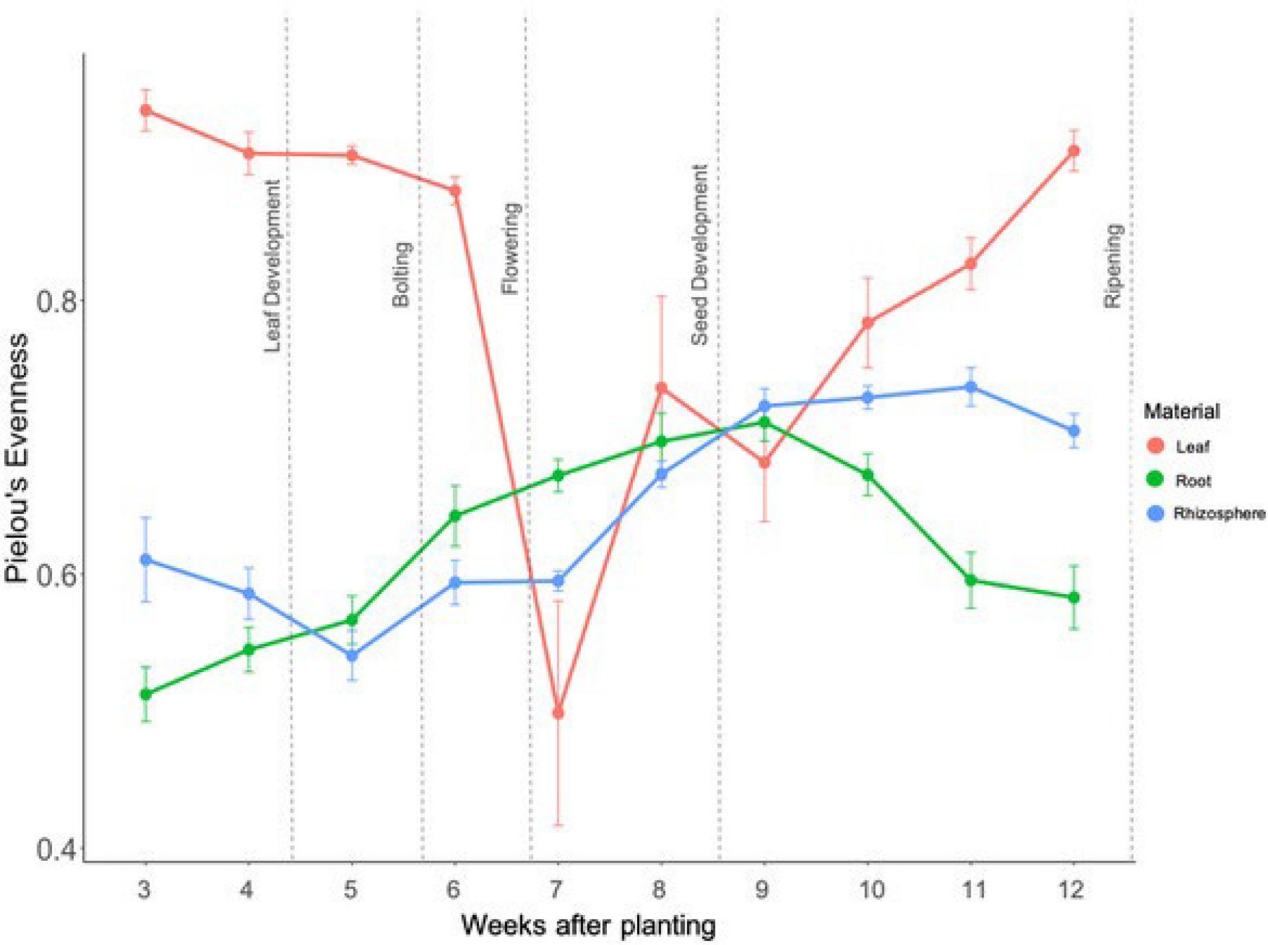
Pielou’s evenness for the leaf (red), root (green), and rhizosphere (blue) over the 10-week sampling period. Each point represents 27 samples, and the error bars are the standard error. Growth stage is indicated by the dashed lines. The larger the number, the more even the community.

βNTI values followed similar patterns as NRI and NTI values (Fig. 3). After week five, from flowering to ripening, rhizosphere βNTI was greater than −2 (*P* > 0.001), indicating homogenous selection was occurring. Root βNTI values only differed from the null hypothesis during week nine (*P* > 0.01), indicating homogenous selection was occurring during this week, but not during previous weeks. Similarly, leaf βNTI values only differed from the null hypothesis (*P* > 0.001) during week six or flowering; however, unlike the root and rhizosphere, the leaf βNTI was less than +2, which suggests heterogenous selection.

**FIG 4:**
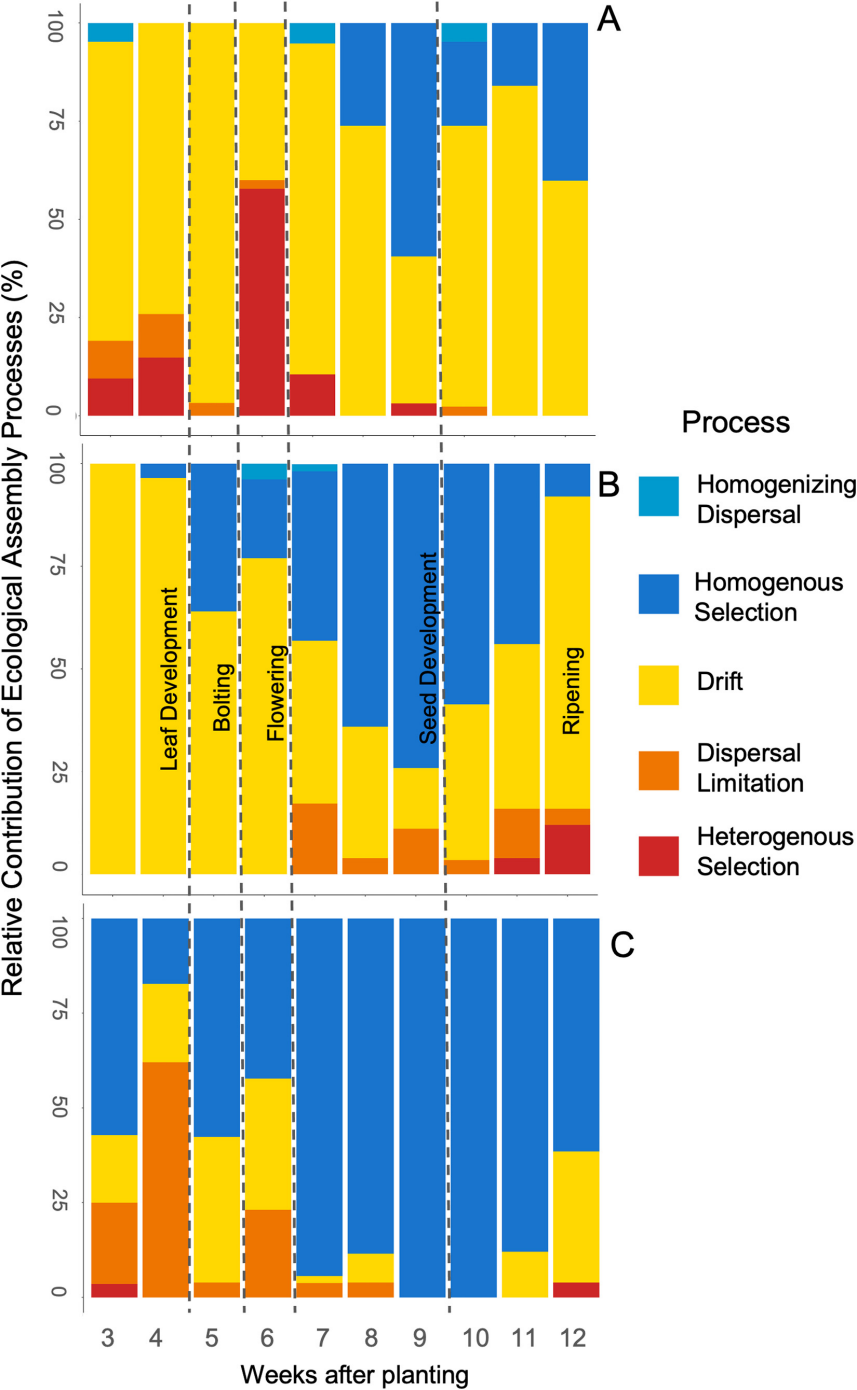
Ecological assembly processes in the bacterial communities present in the leaf (A), root (B), and rhizosphere (C) across all 10 sampling weeks. Deterministic processes were classified as heterogenous selection (βNTI > 2) or homogeneous selection (βNTI < −2). Stochastic processes were classified as homogeneous dispersal (|βNTI| < 2 and RC_bray_ < −0.95) or dispersal limitation (|βNTI| < 2 and RC_bray_ > +0.95). Pairwise observations within the confines of |βNTI| <2 and |RC_bray_| <0.95 were classified as drift/diversification. Growth stage is indicated by the dashed lines.

The primary assembly process in leaves was drift/diversification (Fig. 4A) with only weeks six and seven not being dominated by drift/diversification. Interestingly, after week seven, selection in the leaves moved from heterogenous selection to homogeneous selection. Drift also dominated bacterial community assembly in the root until week seven when the dominant process became homogeneous selection (Fig. 4B). Homogeneous selection remained the dominant process until week 12, when drift dominated again. Homogeneous selection was the dominant process in all weeks in the rhizosphere with the exception of weeks four and six (Fig. 4C). Rhizosphere bacterial communities experienced a noteworthy amount of dispersal limitation, which occurred in weeks three, four, and six, with dispersal limitation as the dominant process during week four (62%). Dispersal limitation was seen in the roots, but this process made up less than 10% every week except weeks six, seven, and nine.

**FIG 5:**
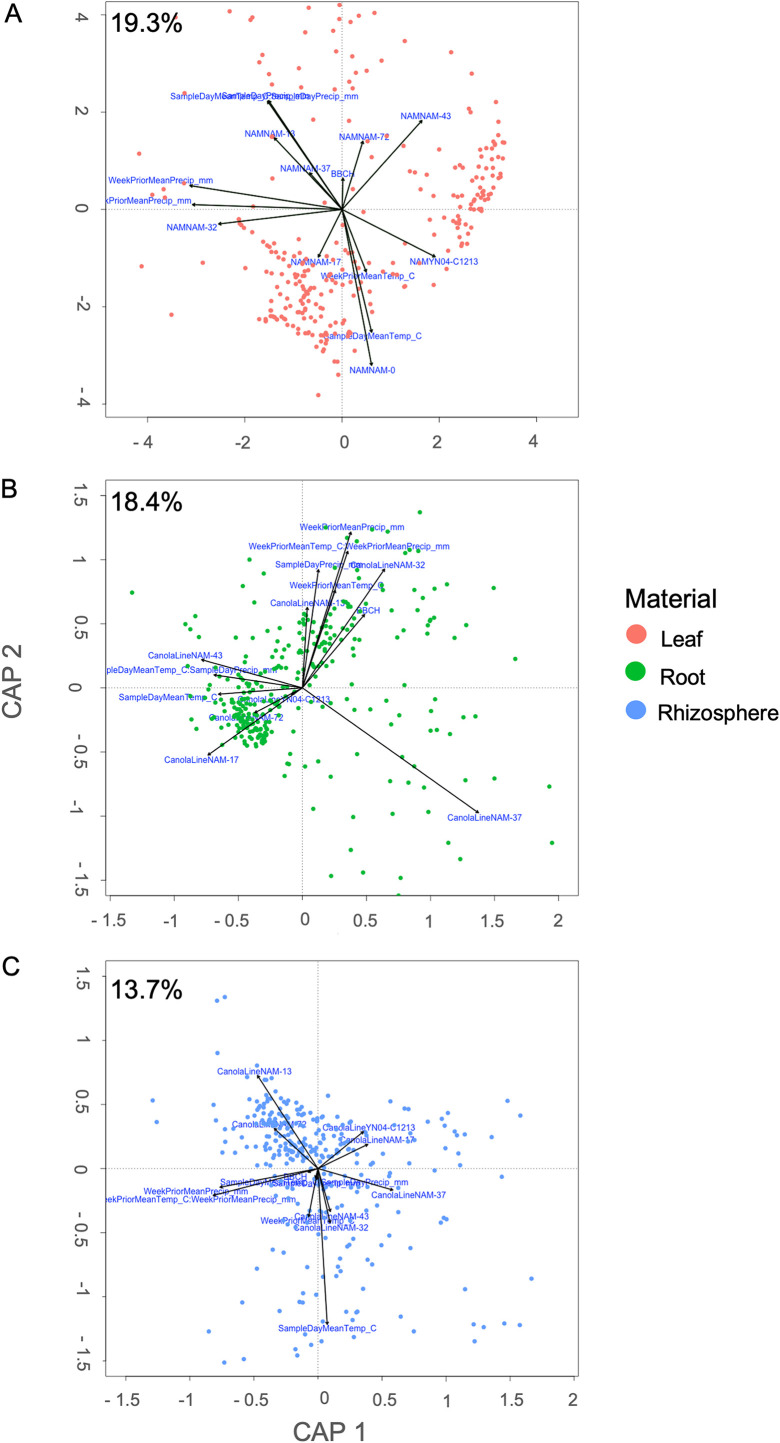
Distance-based redundancy analysis (dbRDA) of the weighted UniFrac distances (A) across the entire 10-week sampling period, constrained by BBCH (p = 0.001) stage × B. napus line (NAM) (not significant) (biplots). The amount of variation explained by the constraints is listed in the lefthand corner. Points are colored based on B. napus growth stage and correspond with the colors in Figure 1 with the leaf in red (A), the root in green (B) and the rhizosphere in blue (C).

To assess which environmental factors could be acting as abiotic filters causing homogenous selection, distance-based redundancy analyses (dbRDAs) were done on the leaf, root, and rhizosphere (Fig. 5) and were constrained by BBCH, prior week mean temperature and precipitation, sampling day mean temperature and precipitation, and NAM line. These filters captured the most variation in the leaf (19.3%) (Fig. 5A), followed by the root (18.4%) (Fig. 5B), with the smallest amount of variation explained in rhizosphere communities (13.7%) (Fig. 5C). Interestingly, while capturing a decent amount of the variation in each plant compartment, none of the factors were significant, nor did the amount of variation captured account for the high levels of deterministic selection seen, especially in the rhizosphere. This suggests the presence of a high number of unmeasured filters, which could be both biotic (inter- or intraspecies interactions) or abiotic (soil factors, relative humidity, etc.).

## DISCUSSION

The root is generally thought to be a highly selective environment (25, 27); however, deterministic selection accounted for more than 50% of the community assembly processes in only 3 out of the 10 weeks, which was not what we hypothesized. Root communities were not more or less clustered than expected by chance and NRI assessments, and strong selection processes were not occurring (βNTI), suggesting that root selection processes are not as strong as previously thought. Using a different approach, i.e., dbRDA, we came to the same conclusion. If the selection processes were primarily deterministic, as we hypothesized, the root assembly processes would have been like the processes observed in the rhizosphere. One reason the root may have been seen as a highly selective environment is because it is consistently less diverse than the rhizosphere soil (25, 28, 29). Our work suggests that this lack of diversity found in the root, relative to the rhizosphere, may arise from the priority effect or competitive exclusion. When a bacterial species can establish itself in or on the root, it could maintain that niche solely through competitive exclusion (30), not allowing more bacterial species to establish and increase diversity (31). If competitive exclusion is the primary reason roots lack diversity, then it would follow that the main community assembly process is drift/diversification, as the community would not change significantly throughout the growing season once the species has established and excluded others. Alternatively, the stable root community could be an example of the priority effect where the order and timing of arrival dictates the species composition of the root (32). If assembly in the root is being affected by the priority effect, the dominance of *Gammaproteobacteria* in the root could be an indication of this. *Gammaproteobacteria* appeared quickly, and its relative abundance did not change much over the 10-week sampling period. Most likely, the stable root community and the predominance of drift as the main assembly process is a combination of both competitive exclusion and priority effects.

The leaf is a harsh environment with high prokaryotic mortality and daily disturbance events from changes in temperature, moisture, and UV radiation (33, 34). Given these difficult conditions, it follows that the major selection pressure is the neutral process of drift/diversification rather than a more plant-driven, deterministic process. Given these severe conditions, it could be possible that no single process was able to dominate due to the high mortality rates and frequent disturbance. Temperature and precipitation accounted for more variation in the leaf community than the root and rhizosphere. Both precipitation events, as well as large temperature fluctuations, would be recurrent disturbance events for the leaf community, causing stochastic processes to dominate, as deterministic processes would be halted. Additionally, the root and rhizosphere is more protected from these recurrent disturbances, which would allow for deterministic processes to continue, which is what was observed.

The rhizosphere effect has been well documented (25, 35, 36) wherein the rhizosphere exhibits changes in bacterial richness compared to the bulk soil. Given the rhizosphere effect is consistent and drastic, there must be deterministic selection processes at work. We saw this reflected in the root where homogeneous selection comprised more than 50% of the selection processes for all weeks except four. In fact, in weeks 9 to 11, homogeneous selection comprised almost all of the selection processes occurring in the rhizosphere. The dominance of homogenous selection could have been caused by the larger root system, which exerted more selection pressure; both of which are correlated with growth stage, which has been documented previously (37). The increase in beneficial bacteria during and after flowering has been documented (38), so the B. napus plants are likely selecting for beneficial species here to increase seed set and ripening. During seed development and ripening, the plant likely undergoes an increased demand for water and nutrients. To meet these demands, the rhizosphere community would have to shift in order to increase nutrient cycling; hence, the dominant deterministic process is homogeneous selection.

One of the hypotheses of this study was that assembly processes would vary with the B. napus (NAM) line, but we were not able to show this. The NAM lines selected for this study were chosen to emphasize differences in various characteristics in hopes of understanding how NAM line shaped the microbiome (39). Despite this careful selection, NAM line had the smallest effect on assembly processes after plant compartment and growth stage. In the rhizosphere, where NAM line had the most consistent effect, the influence of line was not consistent throughout the growing season, suggesting that it did not have a stable influence on the rhizosphere. This is contrary to other studies, which have shown a large effect of plant line on microbial community structure (10, 26). The lack of line differences could be a specific effect of B. napus. Previous work on these same NAM lines has shown that there is no consistent effect of NAM line on phyllosphere bacterial communities nor on the seed microbiome (37, 38). Copeland et al. (3) did not note any effect of canola line on the phyllosphere or rhizosphere as well. Only genetically modified B. napus demonstrated line-level differences in the microbiome, but these did not persist between growing seasons (38, 40). This suggests that for the microbiome of canola, environment and plant growth stage will impact microbial community assembly processes more than differences in canola line.

Growth stage consistently accounted for differences in NTI, NRI, and βNTI (Table 1; Table S4 in the supplemental material), in contrast with variable B. napus line (NAM) influence. Growth stage effect outweighs that of NAM lines that are independent of growth stage alterations. Plants undergo large physiological shifts throughout their life cycles (41–43), which then correspond to changes in the plant microbiome (3, 8, 25, 28, 34). Changes in community assembly processes caused by shifts in plant phenology that result from breeding selection would change not only the composition of the plant-associated communities through deterministic selection. However, shifts in phenology could also change the community dynamics, as one species may have an advantage over other species under these new selection pressures. These changes could alter the benefits that plant-associated communities confer and open a route for more successful microbiome manipulation.

Plant breeders manipulate plant phenology, or growth stage (44), which is the largest determinant of bacterial community assembly processes on B. napus. Manipulating plant phenology, as well as the environmental conditions through inputs, has been suggested as a means of engineering more robust plant microbiomes (2). Periods of time when the microbiome is undergoing strong selection will make good targets for microbiome engineering, as strong selection likely means the plant is selecting for the most fit microbial communities. If the breeding goal is disease reduction, given that most canola diseases are transmitted aerially, it would be wise to focus on the leaf microbiome manipulation. The leaf bacterial community reaches maximum diversity and experiences the strongest selection during the flowering period. Any efforts to manipulate the bacterial microbiome on the leaf should be done before or during when the plant flowers; alternatively, the flowering period could be extended to cultivate more of these beneficial bacteria. Similarly, if the breeding target is larger yields, then focusing on the rhizosphere communities after flowering would likely be the most beneficial. The rhizosphere communities are undergoing strong selection after flowering, which could mean the plant is selecting for beneficial relationships to improve seed production and ripening. Focusing breeding efforts on this time period could impact these processes. Additionally, further study needs to be done on the root exudation patterns occurring during the seed development and ripening periods to determine why the selection pressure is highest during these periods. Root exudation patterns could then serve as a mechanism to engineer beneficial root and rhizosphere communities. A better understanding of the assembly processes of plant microbiomes will allow for the most targeted manipulation and hopefully lead to more robust microbiomes, which can improve agricultural sustainability.

## MATERIALS AND METHODS

### Field collections.

Briefly, in this experiment, we collected samples from eight lines of B. napus plus three random duplicates weekly (*n* = 27) for 10 weeks for a total of 270 samples over the growing season. Additional sequencing samples (*n* = 37) from roots and rhizosphere samples arose from extraction duplicates, PCR duplicates, and sequencing duplicates. In May 2017, eight lines of B. napus (39, 45, 46) were seeded at the Agriculture and Agri-Food Canada (AAFC) research farm outside Saskatoon, Saskatchewan, Canada (52.1718°N, 106.5052°W). These lines of Brassica napus are part of the AAFC canola breeding program created by nested associating mapping, referred to as NAM lines (45). They differed by seed origin and color, fiber content, erucic acid content, and seed glucosinolate levels (Table S1 in the supplemental material). Due to the low erucic acid content, several of these lines are not canola but remain under the B. napus classification. Bazghaleh et al. (47) described the experimental design extensively, but briefly, the experiment was a randomized complete block design consisting of three replicate blocks (6.1 m long by 1.8 m wide) with each B. napus line arranged randomly within each block. All lines were planted on 29 May 2017. The site received 127.9 mm of precipitation throughout the growing season with a mean air temperature of 16.4°C. Both the mean temperature and precipitation were slightly below average for the region. Leaf, root, and rhizosphere samples were collected from each of the eight lines in each block every week for 10 weeks beginning on 20 June 2017 until 22 August 2017. The collections began 3 weeks after planting when the plants were at leaf stage 4 to 6. Root and rhizosphere samples were collected from the same individual plant; however, due to the destructive sampling methods, leaf samples were collected from different plants within the plot.

Root and rhizosphere samples were collected by combining three canola plants from each plot using a sterilized trowel to a depth of approximately 10 cm with a diameter of 15 cm. Plants were extracted down to tap root depth, typically between 5 and 15 cm below surface, and lateral roots in the soil volume occupied by the plant extracted. Lateral roots dominate nutrient acquisition and comprise most of the root surface. A composite of three plants was sampled due to the need for excess root and soil sample material for downstream analysis. Edge rows were avoided to avoid possible contamination with other lines or weeds. Roots with attached rhizosphere soil were placed in a bag, closed, and placed on ice. All samples were stored at 4°C until processing (at most, 24 h). Upon processing, aboveground material was removed, and soil not attached to the roots was collected and stored at −80°C for further analysis. The roots, with adhering rhizosphere soil, were then transferred to a flask containing 100 mL of sterile 0.05 M NaCl buffer and shaken at 180 rpm for 15 min. After shaking, the roots were removed, rinsed with deionized water, and weighed. A subsample of root material was taken from random parts of the root to ensure a random sample, using a flame-sterilized scalpel, and frozen at −80°C for later DNA extraction. The buffer and soil mixture were transferred to centrifuge tubes and centrifuged at 5,000 rpm for 15 min at room temperature. The pellet containing the rhizosphere soil was transferred to 1.5-mL tubes and frozen at −80°C for future DNA extraction. A total of 27 root and rhizosphere samples (8 lines by three blocks, with 3 randomly selected duplicate biological samples) were collected each week over the 10-week sampling period.

Leaf samples were selected by avoiding leaves with visible signs of disease, insect damage, or senescence. Additionally, plants on the edge of plots were avoided, as these plants were visibly dusty. During flowering, B. napus rapidly drops petals, and leaves with heavy flower contamination were also avoided. During the seed development and ripening stages when leaf senescence was advanced, leaves with large amounts of necrotic tissue were avoided. Leaf samples were placed into sterile Whirl-Pak bags (Nasco, WI, USA) and placed onto ice until they were transferred to the lab (~2 h). Leaf samples from the same NAM line but from different blocks were not combined, and plants were not destructively sampled, as only one or two leaves were sampled. The decision to sample a single B. napus plant, unlike taking a composite of three plants like the root and rhizosphere, was done because of the smaller amount of material needed for downstream analysis. Samples were then returned to the lab and stored at −80°C until further processing. A total of 28 leaf samples (8 lines by three blocks, with 3 randomly selected duplicate biological samples) were collected each week over the 10-week sampling period.

### DNA extraction and amplification.

DNA was extracted from 50 mg root tissue using Qiagen PowerPlant extraction kit (Hilden, Germany) following the manufacturer’s instructions. DNA was extracted from 250 mg rhizosphere soil using Qiagen PowerSoil extraction kit following the manufacturer’s instructions. Frozen, brittle leaves were crumbled manually in the Whirl-Pak, and a 0.05-g subsample was taken and extracted using Qiagen PowerPlant extraction kit following the manufacturer’s instructions. Extraction duplicates where the sample material was weighed and extracted twice were included. All root and rhizosphere samples were spiked with a known concentration (0.3 ng μL^−1^) of Aliivibrio fischeri as an internal standard (48). Initially, this was also done with the leaf samples, but after sequencing, it was found that likely due to the naturally low bacterial abundances on leaves, the majority of samples only contained A. fischeri and little host bacteria. Consequently, leaf samples were reextracted without the spike, which greatly improved bacterial amplification. After extraction, DNA was tested for quantity and quality following the standard Qubit protocol (Thermo Fisher Scientific, Waltham, MA).

Rhizosphere DNA was standardized to 5 ng/μL prior to amplification. Root samples were standardized to 1.5 ng/μL prior to amplification. The V3-V4 region of the 16S rRNA was amplified using the primer set 342F with Illumina adapters (5′-TCGTCGGCAGCGTCAGATGTGTATAAGAGACAGCTACGGGGGGCAG-3′) and the 806R (5′-GTCTCGTGGGCTCGGAGATGTGTATAAGAGACAGGGACTACCGGGGTATCT-3′) (49). The PCR mix (25 μL total) contained 2.5 μL DreamTaq buffer (Thermo Fisher Scientific), 2.5 μL deoxynucleoside triphosphate (dNTP) mix (Invitrogen, Carlsbad, California), 1 μL of each primer, 17.75 μL nuclease-free water, and 2 μL of the standardized template DNA. The PCR conditions were 95°C for 5 min as an initial denaturization, followed by 95°C for 30 s, 54°C for 30 s, 72°C for 30 s for 35 cycles, and a final elongation of 72°C for 7 min. Negative controls and PCR duplicates were included.

Template DNA from leaf samples was standardized to 4 ng/μL prior to amplification. Bacterial diversity in leaves was assessed by amplifying the V4 region of the bacterial 16S rRNA using the primer set 515F with Illumina adapters (5′-TCGTCGGCAGCGTCAGATGTGTATAAGAGACAGGTGYCAGCMGCCGCGGTAA-3′) and the 806R (5′-GTCTCGTGGGCTCGGAGATGTGTATAAGAGACAG GGA CTA CCG GGG TAT CT-3′) (50). The 515F/806R primers were selected after failed attempts to amplify with the same primers as the root and rhizosphere. While there are individual primer biases, the 515F/806R primers were deemed the most suitable replacement primers after many leaf amplification failures, as the 342F/806R primer pair covers the entire fragment length of the leaf primer set (49). The PCR reaction mixture consisted of 7 μL Invitrogen Platinum SuperFi PCR master mix (Thermo Fisher Scientific), 0.1 μL of each primer (10 μM stock), 3 μL (5 μM stock) plastid peptide nucleic acid blocker (pPNA), 2 μL (5 μM stock) mitochondrial peptide nucleic acid blocker (mPNA) (PNA Bio, CA, USA), 10.3 μL nuclease-free water, and 2 μL of the standardized template DNA. PNAs were included to block the amplification of host DNA, plant mitochondria, and chloroplasts, which are a common contaminant from plant tissues (51, 52). The PCR conditions were 95°C for 5 min as an initial denaturization, followed by 95°C for 30 s, 78°C for 10 s, 54°C for 45 s, 72°C for 60 s for 35 cycles, and a final elongation of 72°C for 7 min. Negative controls and PCR duplicates were included.

The PCR product was purified to eliminate primers and impurities using a 1:1 ratio of NucleoMag NGS Clean-Up and Size Select kit (D-Mark Biosciences, Scarborough, Ontario). Randomly selected technical duplicates were included during DNA extraction, amplification, and sequencing stages adding in 56 duplicates, bringing the total sample size up to 326. After purification, samples were indexed following the Illumina protocol, purified again to remove excess index primers, quantified and standardized to 4 nM, and pooled. Pooled libraries were then sequenced using the Illumina MiSeq platform using V3 chemistry. Leaf samples were sequenced separately from root and rhizosphere samples. A total of 307 root, 307 rhizosphere soil, and 326 leaf samples were sequenced. Leaf sequencing runs included more technical duplicates than root/rhizosphere runs to ensure amplification due to previous sequencing failure. Quality assurance/control samples included field duplicates, DNA extraction duplicates, library preparation duplicates, and sequencing duplicates.

### Data processing.

A total of 12,813,586 reads were produced for rhizosphere samples with an average of 41,874 per sample. For roots, a total of 12,473,911 reads were produced with an average of 24,584 reads per sample. For leaves, 10,839,325 reads were produced with an average of 18,186 reads per sample. Sequences were imported into QIIME2 v. 2019.1 (53), and primers were removed using cutadapt v. 2020.2.0 (54). Reads were then processed into amplicon sequence variants (ASVs) (55), and chimeras were removed using Deblur (56), resulting in 1,968 ASVs for leaves, 8,987 ASVs for rhizosphere samples, and 4,542 ASVs for root samples. ASVs were classified using a 342F/806R-trained (root/rhizosphere) or a 515F/806R-trained (leaves) V3/V4 SILVA 132 database (57). For leaf samples, host mitochondria and chloroplasts were removed after classification. Host DNA ranged from 6% to 100% of the read in each sample with an average of 32% across samples. Nine samples consisted of entirely host reads and were eliminated from downstream analysis. Mitochondria and chloroplasts were also removed after classification for root and rhizosphere samples; however, they comprised a very low percentage of the overall reads. Reads classified as archaeal, eukaryotes, or unassigned at the kingdom level were removed from all samples but were not abundant overall. The abundance and taxonomy tables produced in QIIME2 were exported to BIOM format (58) for processing in R v. 3.5.3 (54). ASVs that were only represented once in the entire data set or with a sum of zero were removed. Phylogenetic trees were created using the fragment insertion method in QIIME2 (57). Root and rhizosphere abundances were standardized to the Aliivibrio fischeri spike.

### Statistical analysis.

Each plant compartment represents a very different habitat, and consequently, the bacterial communities in each will experience different assembly processes. Due to this and the necessary use of different primer sets, each plant compartment was analyzed separately, and no direct comparisons were made between plant compartments. Each analysis was repeated three times for the leaf, root, and rhizosphere communities.

Abundance-based coverage estimate (ACE) and the Simpson index were calculated using the estimate_richness function on phyloseq v. 1.34.0 (59), and Pielou’s evenness was calculated using the vegan package v. 0.5.1 (60, 61). Permutational analysis of variance (PERMANOVA) was performed using the adonis function in the vegan package in R (60). Bray-Curtis distance matrices were calculated among samples from the same plant compartment (e.g., root) for each time point with the phyloseq package (59).

The BBCH scale (BBCH is not an acronym, but the name of the scale) is a scale used to uniformly identify and quantify the phenological stages of plant development, with scales developed for species-specific development (62). All B. napus lines were assigned BBCH weekly using the Canola Council of Canada BBCH guide (63) and averaged. This was done because despite identical planting times, the eight B. napus lines differed in plant development. Sampling weeks 3 and 4 (WAP) took place during the leaf development stage for most B. napus lines sampled, with bolting during week 5. Peak flowering was reached for most lines during sampling week 6, with seed development occurring in the following 2 weeks. The last 4 weeks of sampling were characterized by ripening of the B. napus seed pods.

Community assembly processes were approached using the null model framework (15, 64). Net relatedness index (NRI) was calculated by using the ses.mpd function (abundance.weighted=TRUE) in the picante package v. 1.8.2 (65). NRI is the number of standard deviations that the observed phylogeny differs from the null mean pairwise distance (MPD) after 999 iterations (65). An NRI value of less than −2 indicates that the community is phylogenetically more dispersed than expected, whereas an NRI value of greater than +2 indicates that the community is more phylogenetically clustered than expected. Similarly, nearest taxon indices (NTIs) were calculated using the ses.mntd function (abundance.weighted=TRUE) in the picante package (65). NTI is the number of standard deviations that the mean nearest taxon distance (MNTD) (66) differs from the null MNTD after 999 iterations. An NTI value of −2 indicates that the community is more distantly related than expected, whereas an NTI value of +2 indicates that the community is more closely related than expected. To state it another way, NRI is the mean branch length between all taxa in the phylogeny, whereas NTI reflects the mean distance between a single taxon and its closest genetic relative. While these metrics are similar, NRI is more sensitive to tree-wide trends of clustering and evenness, whereas NTI is more sensitive to these trends closer to the phylogeny tips (the ends of the branches) (66, 67).

**FIG 6:**
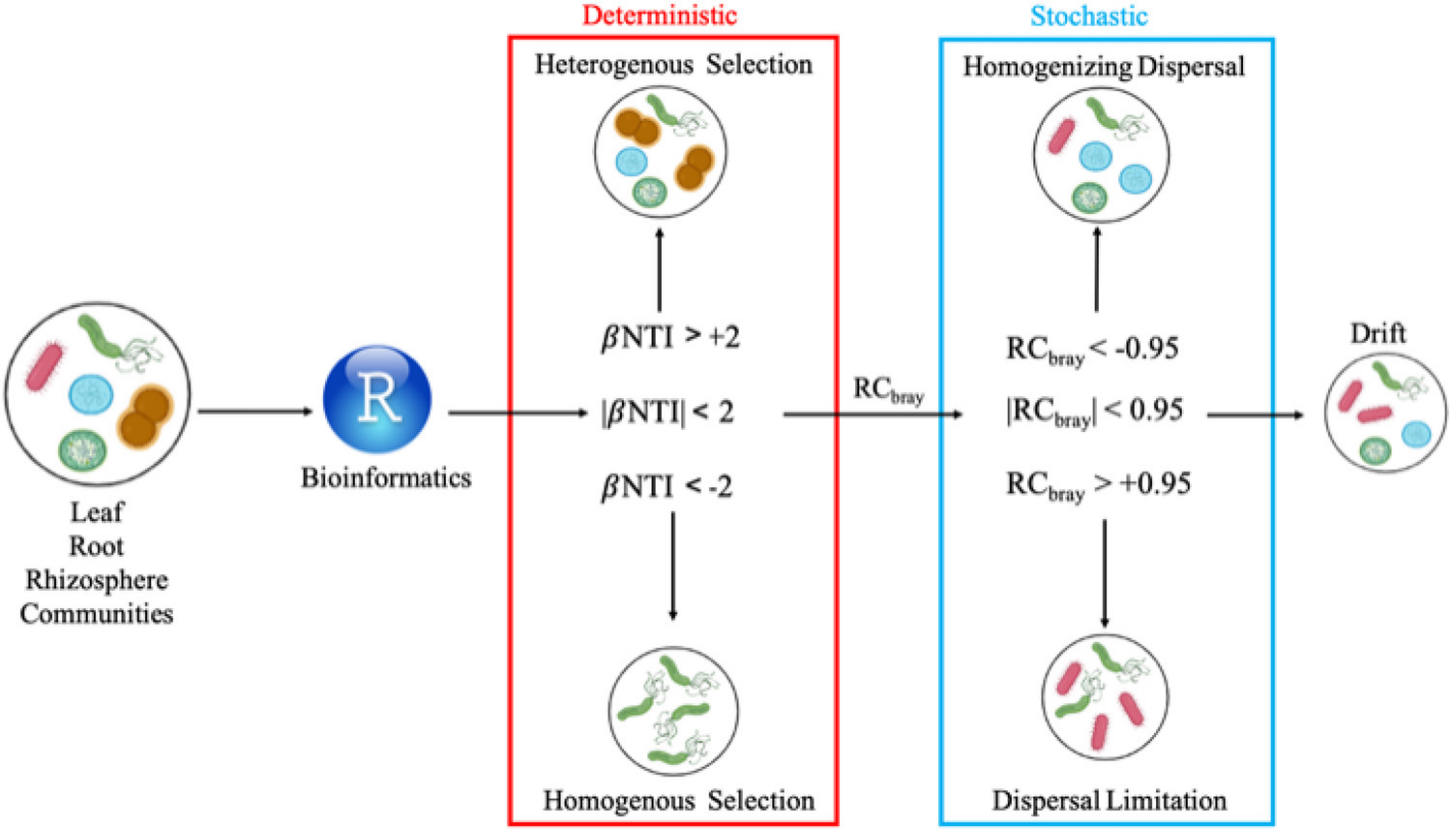
Conceptual diagram of the determination of the assembly processes. Leaf, root, and rhizosphere communities were sampled, sequenced, and processed (see Materials and Methods). Following processing, amplicon sequence variants (ASVs) were imported to R (58). A null model was generated using 999 randomizations from all ASVs present in that community. All pairwise comparisons with a |βNTI| value of >2 are classified as deterministic, with βNTI greater than +2 indicating heterogenous selection and βNTI less than −2 indicating homogeneous selection. Observations with values |βNTI| <2 and RC_bray_ greater than +0.95 were classified as dispersal limitation and observations with values of |βNTI| <2 and RC_bray_ less than −0.95 were classified as homogenizing dispersal. Pairwise comparisons within |βNTI| <2 and |RC_bray_| <0.95 indicated drift or diversification assembly processes were occurring.

Following Stegen et al. (15), selection pressures were quantified using the βNTI metric in the picante package (comdist, abundance.weighted=TRUE) and Bray-Curtis-based Raup-Crick (RC_bray_) in the iCAMP package v. 1.2.9 (14, 65). The βNTI metric indicates the phylogenetic turnover in a given community. RC_bray_ is the probability that a given community is more dissimilar (+1) or less dissimilar (−1) than expected by chance (19). Like the previous metrics, RC_bray_ uses successive iterations to determine these probabilities. βNTI measures the difference between the observed βMNTD and the null βMNTD. Deviation from the null βMNTD indicates that the community is undergoing some level of selection or filtering that is not random (null βMNTD). The null distributions for both metrics were generated weekly for each plant compartment using 999 randomizations. |βNTI| of >2 indicates that deterministic selection dominates community assembly processes at a 5% significance level (14). βNTI values of >2 were classified as heterogenous selection (Fig. 6, red box). βNTI values less than two were classified as homogeneous selection. Observations of |βNTI| <2 indicated predominance of stochastic, rather than deterministic, processes (*P* < 0.025). Pairwise comparisons between βNTI and RC_bray_ were done to determine the stochastic processes dominating bacterial community assembly (Fig. 6, blue box). Observations with values of |βNTI| <2 and RC_bray_ >+0.95 were classified as dispersal limitation, and |βNTI| less than 2 and RC_bray_ less than −0.95 classified as homogenizing dispersal (14, 16, 64, 66). Pairwise observations not having values of |βNTI| of <2 or |RC_bray_| <0.95 were categorized as drift or diversification (Fig. 6). This could indicate that this population is weakly experiencing any of the previously mentioned processes or that the community is undergoing drift (both ecological or genetic), which is the random division, death, or mutation (diversification) of individual community members (68).

To examine which factors influenced deterministic selection processes, distance-based redundancy analysis (dbRDA) (69) was performed on weighted UniFrac distance matrices (70) using the capscale function in the vegan package in R (59). The UniFrac distances were calculated using the phyloseq package (59). UniFrac distances were used to preserve the phylogenetic relationships in the communities. Leaf, root, and rhizosphere dbRDAs were constrained by BBCH, week prior mean temperature and precipitation, sampling day mean temperature and precipitation, and NAM line. These factors were chosen, as they would impact all the measured habitats (the leaf, root, and rhizosphere), whereas soil factors would largely influence the root and rhizosphere, but not the leaf. All code is available at https://github.com/jbell364/Canola-Selection.

### Data availability.

All raw sequence files can be found at the National Center for Biotechnology Information (NCBI) under BioProject accession nos. PRJNA635907 and BioProject PRJNA575004.
