# Supplementary material for: Brassica napus Bacterial Assembly Processes Vary with Plant Compartment and Growth Stage but Not between Lines
Source: Appl Environ Microbiol. 2022 Apr 28;88(10):e00273-22. doi: 10.1128/aem.00273-22 (PMC9128504; doi:10.1128/aem.00273-22)
Supplement: Supplemental file 1 — Fig. S1 and legends of Tables S1 to S5. Download aem.00273-22-s0001.pdf, PDF file, 0.3 MB [file aem.00273-22-s0001.pdf]

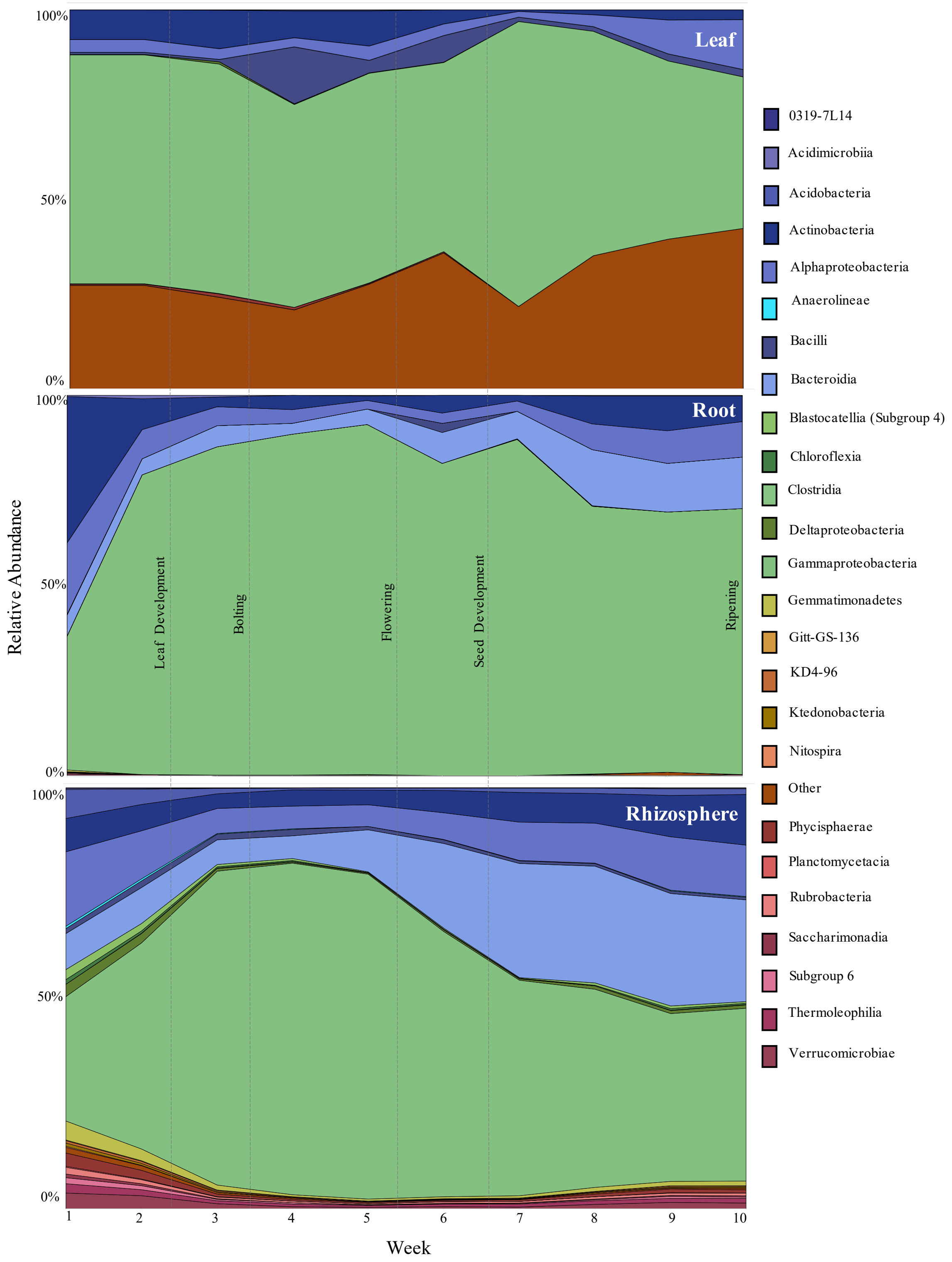

Figure 1: Relative abundance of bacterial communities on the leaf (A), root (B) and rhizosphere (C) over the ten week sampling period. Color represent bacterial classes and the growth stage of *B. napus* is indicated by the dotted line.

Table 1: Weekly diversity indices for the leaf, root and rhizosphere over the ten sampling weeks

Table 2: Weekly PERMANOVAs of the effect of NAM line on leaf bacterial communities.

Table 3: Weekly PERMANOVAs of the effect of NAM line on root bacterial communities.

Table 4: Weekly PERMANOVAs of the effect of NAM line on soil bacterial communities.

Table 5: Two-way ANOVA for the effect of *B. napus* line (NAM) and growth stage (BBCH) on the BNTI values for the leaf, root and rhizosphere over the ten week sampling period. There were no significant interactions so they were not included in the final model.
